# Complex sense-antisense architecture of *TNFAIP1/POLDIP2 *on 17q11.2 represents a novel transcriptional structural-functional gene module involved in breast cancer progression

**DOI:** 10.1186/1471-2164-11-S1-S9

**Published:** 2010-02-10

**Authors:** Oleg V Grinchuk, Efthimios Motakis, Vladimir A Kuznetsov

**Affiliations:** 1 Bioinformatics Institute, 30 Biopolis Str. #07-01, 138672, Singapore

## Abstract

**Background:**

A sense-antisense gene pair (SAGP) is a gene pair where two oppositely transcribed genes share a common nucleotide sequence region. In eukaryotic genomes, SAGPs can be organized in complex sense-antisense architectures (CSAGAs) in which at least one sense gene shares loci with two or more antisense partners. As shown in several case studies, SAGPs may be involved in cancers, neurological diseases and complex syndromes. However, CSAGAs have not yet been characterized in the context of human disease or cancer.

**Results:**

We characterize five genes (*TMEM97*, *IFT20*, *TNFAIP1*, *POLDIP2 *and *TMEM199*) organized in a CSAGA on 17q11.2 (we term this the *TNFAIP1/POLDIP2 *CSAGA) and demonstrate their strong and reproducible co-regulatory transcription pattern in breast cancer tumours. Genes of the *TNFAIP1/POLDIP2 *CSAGA are located inside the smallest region of recurrent amplification on 17q11.2 and their expression profile correlates with the DNA copy number of the region. Survival analysis of a group of 410 breast cancer patients revealed significant survival-associated individual genes and gene pairs in the *TNFAIP1/POLDIP2 *CSAGA. Moreover, several of the gene pairs associated with survival, demonstrated synergistic effects. Expression of genes-members of the *TNFAIP1/POLDIP2 *CSAGA also strongly correlated with expression of genes of *ERBB2 *core region of recurrent amplification on 17q12. We clearly demonstrate that the observed co-regulatory transcription profile of the *TNFAIP1/POLDIP2 *CSAGA is maintained not only by a DNA amplification mechanism, but also by chromatin remodelling and local transcription activation.

**Conclusion:**

We have identified a novel *TNFAIP1/POLDIP2 *CSAGA and characterized its co-regulatory transcription profile in cancerous breast tissues. We suggest that the *TNFAIP1/POLDIP2 *CSAGA represents a clinically significant transcriptional structural-functional gene module associated with amplification of the genomic region on 17q11.2 and correlated with expression ERBB2 amplicon core genes in breast cancer. Co-expression pattern of this module correlates with histological grades and a poor prognosis in breast cancer when over-expressed. *TNFAIP1/POLDIP2 *CSAGA maps the risks of breast cancer relapse onto the complex genomic locus on 17q11.2.

## Background

A *cis*-sense antisense gene pair (SAGP) comprises a gene pair in which the individual genes map to opposite strands on the same DNA locus and are, therefore, transcribed in opposite directions. The corresponding pairs of *cis*-antisense transcripts are mRNAs that are at least partially complementary to each other. *Cis*-antisense mRNAs that are naturally transcribed from a SAGP are known as naturally occurring sense-antisense (SA) RNAs.

Studies have shown that changes in the transcription of SAGPs could be implicated in pathological processes such as some cancers and neurological diseases [1-3]. For example, it was shown experimentally in leukemia cells that genes BAL1 and BBA, which form a SAGP, are bi-directionally transcribed and concordantly expressed due to INF-gamma induction and that their products can directly interact at the protein level [4]. Previously we reported that 12 high-confidence SAGPs pairs are concordantly regulated in human breast cancer tissues [5]. Among these, two pairs (*RAF1/MKRN2 *and *CKAP1/POLR2I*) are constitutively co-regulated in breast tumors of different genetic grades (G1, G1-like, G3-like, and G3), while the co-expression of the *CR590216/EAP30 *SAGP is observed specifically in G3 genetic grade.

In mammalian genomes, SAGPs can be organized in more complex sense-antisense gene architectures (CSAGAs) in which at least one gene shares loci with two or more antisense partners [6-8]. Many dozens of CSAGAs can be found in the human genome - [8-10]; therefore, it is an intriguing speculation that not only SAGPs, but also CSAGAs, as integrated modules, may play important roles in human diseases, including cancer. In this regard, the study of the co-regulatory profiles of genes in the same CSAGA and, possibly, between different CSAGAs or other transcriptional modules would shed new light on the complex nature of the entire transcriptome.

There are many oncogenes on chromosome 17, although the localization of these genes is not uniform. For example, according to Cancer Genetics Web http://www.cancer-genetics.org, the oncogenes *TAF2N*, *NF1 *and *THRA *are located on 17q11.1-q12. *ERBB2 *(*Her-2/neu*), a well-known oncogene, is located on 17q12. The gene BIRC5 on 17q25.3, which encodes the apoptosis inhibitor survivin, co-amplifies with ERBB2 and correlates with high histological grade and a poor prognosis in breast cancer when overexpressed [11]. Many other genes located close to *ERBB2 *on 17q12 could be over-expressed or/and amplified and are known or suspected to play a role in carcinogenesis, specifically, breast carcinogenesis. Previous studies have demonstrated that the negative effect on the prognosis of breast cancer attributed to *ERBB2 *amplification could, in fact, be due to co-amplification of the region adjacent to *ERBB2 *[12]. The *ERBB2 *gene and its neighbour genes could be amplified and over-expressed in 25% of invasive breast carcinomas [13,14]. In general, *ERBB2 *amplification and over-expression confers an unfavourable prognosis, although its significance is less than that of the traditional prognostic factors of stage and grade. It also seems that the prognosis and response to therapy varies considerably within the spectrum of *ERBB2*-amplified breast carcinomas, indicating that they are biologically heterogeneous [14].

CSAGAs and their association with human cancers in the regions outside of the *ERBB2 *amplicon core region in 17q12 [15] have not been studied. It is possible that a high diversity of breast cancer cell subtypes could be associated with active chromatin regions on 17q that are different from the *ERBB2 *amplicon region. We focused on a CSAGA located on 17q11.2 composed of five genes and including the convergent SAGP *TNFAIP1/POLDIP2*. We assume that novel CSAGAs important in breast cancer development could be found in highly unstable regions of the genome and that these complex architectures could play significant roles in transcription control, resulting in cancer phenotypes and impacting patient survival.

## Methods

### Patients, tumor specimens, cell lines and microarray data

Clinical characteristics of breast cancer patients and tumor samples from two independent cohorts (Uppsala and Stockholm) have been published previously [16]. The Stockholm cohort comprised *K*_*s *_= 159 patients with breast cancer, who were operated on in the Karolinska Hospital from 1 January 1994 to 31 December 1996 and identified in the Stockholm-Gotland breast Cancer registry [16]. The Uppsala cohort involved *K*_*u *_= 251 patients representing approximately 60% of all breast cancers resections in Uppsala County, Sweden, from 1 January 1987 to 31 December 1989. Information on patients' disease-free survival (DFS) times/events and the expression patterns of approximately 30,000 gene transcripts (representing *N *= 44,928 probe sets on Affymetrix U133A and U133B arrays) in primary breast tumors was obtained from the National Center for Biotechnology Information (NCBI) Gene Expression Omnibus (GEO) (the Stockholm data set ID is GSE4922; the Uppsala dataset ID is GSE1456). The microarray intensities were MAS5.0 calibrated and the probe set signal intensities log-transformed and scaled by adjusting the mean signal to a target value of log500. For association studies of DNA copy number and gene expression we utilized single nucleotide polymorphism (SNP) copy number microarray data for 46 breast cancer cell lines [12] (GEO data set IDs: GSE13696-GPL2004, and GSE13696-GSL2005) as well as gene expression profiling of 51 human breast cancer cell lines downloaded from the GEO: GSE12777.

### Correlation analysis

Our primary goal is to identify whether the set of genes composing the *TNFAIP1/POLDIP2 *CSAGA forms a significant cluster. First, we estimate Pearson correlation coefficients among these genes in the two large cohorts and subsequently test whether their matrices are significant at level *α *= 1%. Using Pearson correlation coefficient requires that the data are normally distributed. To show that our data satisfy this assumption, we run Kolmogorov-Smirnov test for Normality with Lilliefor's P value correction. Additional file 1 contains Supplementary Tables S1a-d shows the results for each grade in each cohort. Evidently, our data can be thought to come from a Gaussian distribution.

Then, we derive a correlation matrix of the form:

where *r*_1*p *_denotes the Pearson correlation coefficient between Affymetrix probesets 1 and *p*, estimated from the microarray expression data, and *p *is the total number of probes in the prospective cluster.

To test the significance of the *R *matrix, we used a bootstrap version of Bartlett's statistical test [17]. The bootstrap Bartlett test evaluates the significance of the hypothesis H_0_: *R*_*p*×*p *_= *I*_*p*×*p*_, where *R*_*p*×*p *_is the p × p correlation matrix and *I*_*p*×*p *_is the corresponding p × p identity matrix. Under the null hypothesis, there is no significant correlation among these probes, whereas rejection of H_0 _at *α *= 1% is an indication of a cluster. For the *p *genes of the correlation matrix one needs to compute the statistic:

where *N *is the sample size (number of patients in each cohort), *p *is the number of variables (probes) and |*R*| is the determinant of the sample correlation matrix. This quantity is distributed approximately as *χ*^2 ^with 1/2 *p(p-1) *degrees of freedom. To test the significance of the statistic, we draw B = 5,000 samples of *p *neighbouring genes (genes located close to each other) at random from the set of 44,928 genes and estimate Bartlett's t-test, *T*^*b*^, for each of the B = 5,000 draws. The corresponding bootstrap P value is estimated as:

where *T*^*b *^denotes the bootstrap test statistic of the b^*th *^draw. Similar bootstrap approaches have been discussed in [18].

### Comparison of correlation matrices

We would like to show that the genes in the *R *matrix form a significant, tight cluster that cannot be re-produced in the neighbourhood. For our analysis we use Box's M test [19], which evaluates the significance of the hypothesis H_0_: *R*_*p*×*p *_=  where *R*_*p*×*p *_is as before and  is a *q *× *q *correlation matrix of the neighbouring genes (in our case *q *>*p*). Note that *R *and R* should have equal dimension but the correlation coefficients *r*_*a*,*b *_(*a*, *b *= 1,.., *p*) of *R *and  of R* can be estimated from unequal sample sizes *v*_1 _and *v*_2 _and used to estimate Box M statistic as:

|*S*_1_| is the determinant of the variance-covariance matrix of our prospective gene cluster (corresponding to the *R*_*p*×*p *_correlation matrix), |*S*_2_| is the determinant of the variance-covariance matrix of the neighbouring group of genes (corresponding to the  correlation matrix) and |*S*_*pool*_| is the pooled sample variance/covariance matrix estimated as:

Box [19] gave *χ*^2 ^and F approximations for the distribution of M (an exact test does not exist). Notice that in our case *v*_1 _= *v*_2 _but the dimensions of *R *and R* differ. To compare *R *and R* we form all possible  matrices and compare each one with *R*_*p*×*p *_using Box M test. Then we average over the estimated P values. It is possible that our approach introduces some bias in the comparison. However, as we will see later, the difference between the two compared matrices is large enough to safely conclude for their statistical difference.

### Survival Analysis Based on Genes and Gene Pair Expression Patterns

This analysis involves testing whether the prospective gene cluster contains *survival significant genes *and *gene pairs*. As survival significant we consider the genes whose expression levels are significantly correlated with survival times/events. The approach that we follow is called data driven grouping and has been extensively discussed in [20]. Here we briefly describe the idea of the method.

We assume a microarray experiment with *i *= 1, 2,..., *p *genes, whose log-transformed intensities are measured for *k *= 1, 2,..., *K *patients. Associated with each patient are continuous clinical outcome data (Disease Free Survival time (DFS), *t*_*k*_, defined as the time interval from surgery until the first recurrence (local, regional, distant) or the last date of follow-up), and a nominal (yes/no) clinical event *e*_*k *_(e.g., occurrence of tumor metastasis at time *t*_*k*_). Each patient is assigned to low- or high- risk groups according to:

where *c*^*i *^denotes the cut-off of the *i*th gene's intensity level. Motakis et al. [20] showed how to estimate this cut-off from the data by maximizing the distance of the Kaplan-Meier survival curves of the two patients groups. This algorithm is called one-dimensional data-driven grouping (1D DDg). The clinical outcomes/events are subsequently fitted to the patients' groups by the Cox proportional hazard regression model [21]:

where *h*^*i*^_*k *_is the hazard function and *α*_*i*_(*t*_*k*_*) = *log *h*^*i*^_0_(*t*_*k*_*) *represents the unspecified log-baseline hazard function; *β *is the 1 × *p *regression parameter vector; and *t*_*k *_is patient survival time. To assess the ability of each gene to discriminate the patients into two distinct genetic classes, the Wald P value of the *β*_*i *_coefficient of the Cox proportional hazard regression model [21] is estimated by using the univariate Cox partial likelihood function, estimated for each gene *i *as:

where *R*(*t*_*k*_) = {*j*: *t*_*j *_≥ *t*_*k*_} is the risk set at time *t*_*k *_and *e*_*k *_is the clinical event at time *t*_*k*_. The actual fitting of the Cox model is conducted by the *survival *package in R http://cran.r-project.org/web/packages/survival/index.html. The genes with the significant *β*_*i *_Wald P values are assumed to have better group discrimination ability and are thus called *"highly survival-significant genes"*. These genes are selected for further confirmatory analysis or for inclusion in a prospective gene signature set.

The proposed dichotomization of the patients into two groups and the subsequent fit on the Cox proportional hazards model is a strategy that has been followed in the past (for example see [5]) to identify clinical groups of patients. Our data-driven method is an improvement of the mean-based approach [5] as showed in [20]. Data-driven grouping estimates the optimal partition (cut-off) of a single gene's expression level by maximizing the separation of the survival curves related to the high- and low- risk of the disease behaviour. In this sense it does not rely on predefined cut-offs (like mean-based does) and, more importantly, it is able to identify several survival significant genes that cannot possibly be found by other methods. Our technique has the potential to be a powerful tool for classification, prediction and prognosis of cancer and other complex diseases. Extensive discussion and evaluation of our method can be found in [20].

A similar approach is applied to identify synergistic survival-significant gene pairs using the two-dimensional data-driven grouping method of Motakis et al. [20]. Briefly, for a given gene pair *i*, *j *with individual cut-offs (identified from the one- dimensional data-driven grouping) *c*^*i *^and *c*^*j*^, *i *≠ *j*, we may classify the *K *patients by seven possible two-group designs.

**Figure 1:**
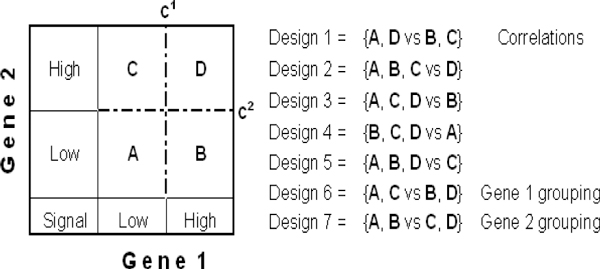
**Grouping of a synergetic gene pair (genes 1 and 2 with respective cutoffs c^1 ^and c^2^) and all possible two-group designs (Designs 1-7)**.

Figure 1 indicates the regions where patients' gene intensities [*y*_*i*,*k*_, *y*_*j*,*k*_] are plotted; note that "A", "B", "C" and "D" are defined by the conditions A: *y*_*i*,*k *_<*c*^*i *^and *y*_*j*,*k*_<*c*^*j*^; B: *y*_*i*,*k *_≥ *c*^*i *^and *y*_*j*,*k *_<*c*^*j*^; C: *y*_*i*,*k*_<*c*^*i *^and *y*_*j*,*k *_≥ *c*^*j*^; D: *y*_*i*,*k *_≥ *c*^*i *^and *y*_*j*,*k *_≥ *c*^*j*^. For each *i *and *j *pair (*i*, *j *= 1,..., *p*), group the patients according to each of the seven designs shown in Figure 1 (using individual gene cut-offs), fit the Cox model:

for each design and estimate the seven Wald *P value*s for  (*d *= 1,..., 7 corresponds to designs). Provided that the sample sizes of the respective groups are sufficiently large and the proportionality assumption of the Cox model is satisfied (the ratio of the hazards does not depend on time), the best grouping scheme among the five "synergistic" (designs 1 to 5) and the two "independent" (designs 6 and 7) designs is that with the smallest *P- *value. We perform multiple testing corrections later when the best designs and P values have been collected for each gene pair. At that stage we will identify the truly prognostic significant genes by minimizing the number of false positives. The procedure we will apply is the False Discovery Rate [29]. Extensive discussion is provided at the paragraph 'Survival analysis of SFGM genes and their closest neighbours in breast cancer patients'.

The correlation and survival analyses were conducted in R http://cran.r-project.org/ using software developed by our group. All our programs are available upon request.

## Results

### Identification of the co-expressed *TNFAIP1/POLDIP2 *sense antisense gene pairs

**Figure 2:**
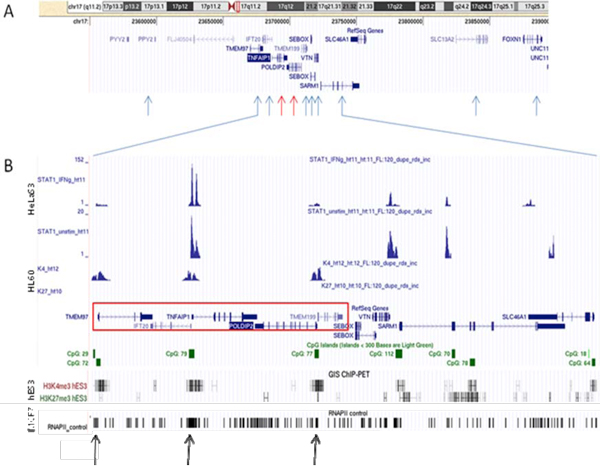
***TNFAIP1/POLDIP2 *complex sense antisense architecture mapped onto the genome (UCSC genomic browser)**. **A **- *TNFAIP1/POLDIP *SAGP (red arrows) and *TMEM97/IFT20 *SAGP (two next closest blue arrows on the left) with seven other genes included in the analysis (blue arrows). **B **- *TNFAIP1/POLDIP2 *complex cis-sense antisense architecture (red box) with different tracks. Small green solid boxes represent CpG islands, and green transparent boxes represent regions of enrichment of potential miRNA regulatory target sites. ChIP-Seq tracks represent regions of DNA binding by STAT1 (human cervical cancer HeLaS3 cell line [42]), ChIP-PET-defined histone trimethylations H3K4me3 and H3K27me3 (promyelocytic leukemia cell line HL60 [40]) and ChIP-seq-defined RNA polymerase II binding (breast cancer cell line MCF7 [44]). Black arrows at the bottom indicate the direct evidence of transcription activation of the *TNFAIP1/POLDIP2 *CSAGA in the breast cancer cell line. The GIS ChiP-PET track shows H3K4me3 and H3K27me3 regions mapped on the human genome (embryonic stem cell line hES3 [41]).

Using the high-confidence Affymetrix Chip U133 A&B probesets presented in the APMA database [22], http://apma.bii.a-star.edu.sg/, we selected 156 SAGPs located on chromosome 17 with reliable RefSeq support (those IDs with NM prefixes) for each member of each pair. Each of the genes in these SAGPs was supported by at least 1 Affymetrix Chip U133 A&B probesets. We focused on chromosome 17 because many regions of that chromosome are actively involved in recurrent amplifications during breast cancer development (including the *ERBB2 *amplicon). Using mRNA expression data from the Uppsala and Stockholm cohorts, we calculated Pearson correlations for each pair and identified high-confidence correlated pairs of probesets (*α *= 1%) representing twelve SAGPs. Among these positively- and highly-correlated SAGPs, two convergent SA gene pairs (the *TNFAIP1/POLDIP2 *SAGP and the *IFT20/TMEM97 *SAGP) attracted our attention (Figure 2B) because *TNFAIP1* and *IFT20* also have a common SA overlapping region. Thus, these two SAGPs were in fact the parts of the same complex CSAGA. Our further work was focused on the detailed characterization of this CSAGA.

*POLDIP2 *(NM_015584) encodes a protein that interacts with the DNA polymerase delta p50 subunit and with proliferating cell nuclear antigen (*PCNA*) [23]. Some transcripts of this gene overlap in a tail-to-tail orientation with the gene for tumor necrosis factor alpha-induced protein 1 (*TNFAIP1*; NM_021137). The genes of this pair form a convergent (tail-to-tail) gene orientation topology, share a 376-nucleotide region of their 3'-untranslated regions and are located on human chromosome cytoband 17q11.2. It has been reported that this gene can be induced by *TNF-alpha *[24]. Moreover, the *TNFAIP1 *protein can also directly interact with the *PCNA *protein. The rat TNFAIP1 stimulates polymerase delta activity *in vitro *in a *PCNA*-dependent way [25]. Thus, transcription of *POLDIP2 *and *TNFAIP1 *could be under common control and the products of these genes could be involved in the same pathways.

### Identification of TNFAIP1/POLDIP2 Structural-Functional Gene Module

We identified two SAGPs (*TNFAIP1/POLDIP2 *SAGP and *IFT20/TMEM97 *SAGP) located close to each other on 17q11.2. These SAGPs demonstrated reproducible and significant co-expression pattern in 2 independent cohorts (the Uppsala and Stockholm cohorts) of breast cancer patients. When the genes composing the SAGPs were analyzed as a pair in survival analysis, their co-expression turned out to be survival significant in both cohorts. For TNFAIP1/POLDIP2 SAGP, the P value (and designs) for Stockholm was 4.6E-04 (design = 1) and the corresponding values for Uppsala was 3.1E-07 (design = 1). For IFT20/TMEM97 SAGP, the P value (and designs) for Stockholm was 3.6E-03 (design = 2) and the corresponding values for Uppsala was 1.6E-05 (design = 2). The plots of patients grouping and Kaplan-Meier survival curves are shown in 'Materials and methods' section.

**Figure 3:**
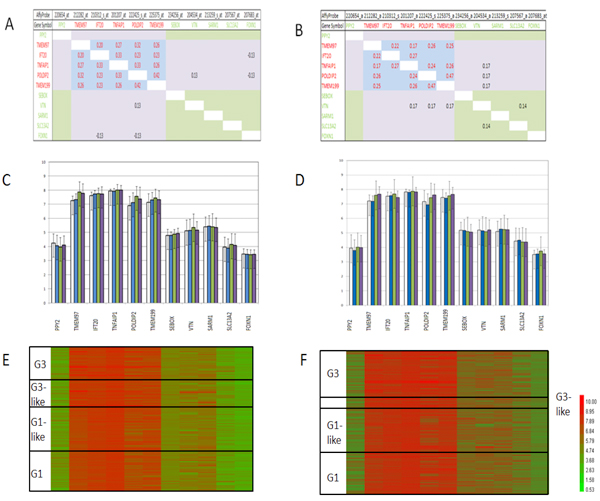
**Members of the *TNFAIP1/POLDIP2 *CSAGA are mutually co-regulated in breast cancer and form a structural--functional gene module**. Correlation matrices visually demonstrate the presence of a characteristic co-regulatory pattern: the co-regulatory area is formed by enrichment of significant Pearson correlation coefficients (*α *= 1%). Members of the *TNFAIP1/POLDIP2 *CSAGA form a SFGM (*blue matrix - *SFGM matrix); the *light green matrix *shows correlations among six 'neighbouring' genes (the 'neighbouring' genes matrix - NG matrix); the *light violet matrix *area marks intergroup correlations between genes of the SFGM matrix and NG matrix.. **A, C **and **E **-- Uppsala cohort; **B, D **and **F **-- Stockholm cohort; **A **and **B **-- correlation matrices, **C **and **D **- individual gene expression in breast cancer patients in different breast cancer grades (G1, G1-like, G3-like and G3 [16]), **E **and **F **- heat map visualization of gene expression in different grades of breast cancer.

Next, we produced correlation matrices of the *TNFAIP1/POLDIP2 *and *IFT20/TMEM97 *SAGPs with seven more neighbouring genes, including the one closest gene (*PPY2*) located centromeric to *IFT20/TMEM97* SAGP and six closest genes (*TMEM199*, *SEBOX*, *VTN*, *SARM1*, *SLC13A2*, *FOXN1*) located telomeric to *TNFAIP1*/*POLDIP2* SAGP. According to the UCSC genomic browser (hg18), the most distant centromeric gene, *PPY2*, is located at a distance not exceeding 80 kb from *TNFAIP1/POLDIP2 *SAGP. The most distant of the telomeric genes, *FOXN1*, is located at a distance of 170 kb (Figure 2A). Affymetrix U133A&B probesets 214283_at (gene *TMEM97*), 229182_at as well as 233531_at and 234060_at (gene *SLC46A1*) were excluded from our analysis due unclear support by the well annotated and reliable RefSeq gene database. Genes (and their Affymetrix probes) in the matrix were placed in the same order as they are located on 17q11.2 in the human genome. Analyzing the correlation matrices of these 11 genes, we discovered that 5 of them are structurally organized as complex sense antisense gene architecture (CSAGA) (Figure 2B). These genes are *TMEM97, IFT20, TNFAIP1, POLDIP2 *and *TMEM199*. Figure 3A, B shows their strong mutual correlation pattern in breast cancer patients in both breast cancer cohorts. The expression levels of each of these five genes in different grades of breast cancer in both cohorts were much higher compared to the 6 centromeric and telomeric neighbours in the chosen genomic window (Figure 3C, D). Also, significant differences in gene expression levels were observed for *TMEM97 *and *POLDIP2 *in different grades of breast cancer in both cohorts (not shown). We performed heat map analysis using Tree View 1.1.3 software [26] - which showed a clear overexpression cluster of the five-gene module compared to its centromeric and telomeric neighbours in both breast cancer cohorts (Figure 3E, F).

The structural backbone of this *TNFAIP1/POLDIP2 *CSAGA is composed of three CpG rich regions representing putative gene promoters (two of which are bidirectional), as well as two intergenic convergent SA overlaps (*TMEM97* and* IFT20*, *TNFAIP1* and *POLDIP2*) with RefSeq support (Figure 2B) and one divergent SA overlap with UCSC support (*IFT20* and *TNFAIP1*) (data not shown).

Based on its structural and expressional integrity, we have termed the *TNFAIP1/POLDIP2 *CSAGA a *TNFAIP1/POLDIP2 structural - functional gene module *(SFGM). For the remaining six genes in the chosen window we use the term 'neighbouring' genes for the convenience of description.

Next, using Bartlett's test [17] and Box's M test [19], we addressed the following questions: first, whether the correlation matrices for the five genes of the *TNFAIP1/POLDIP2 *SFGM (the SFGM matrix) as well as the correlation matrices for the six 'neighbouring' genes ('neighbouring' genes matrix - NG matrix) are statistically significant compared with randomly chosen matrices derived from genes close to each other in the whole genome (Figure 3A, B); and, second, whether SFGM matrices are significantly different from NG matrices. As discussed before our second task involved comparing two matrices of unequal dimension. For this reason, we found all possible 6-genes signatures composed by NG matrices and compared each respective matrix with the SFGM matrix. Then, we averaged the test P values and reported our results in Table 1.

**Table 1:** P values obtained by pair-wise comparisons of matrices for five genes in the SFGM group and six 'neighbouring' genes

Breast cancer grade	SFGM matrix ^1^		NG matrix ^1^		SFGM matrix/NG matix ^2^	
	**U**	**S**	**U**	**S**	**U**	**S**

G3	1.5E-12	2.7E-10	7.6E-01	5.9E-01	1.0E-16	1.0E-16
G3-like	3.4E-11	8.9E-03	2.1E-01	8.8E-01	1.0E-16	1.1E-01
G1-like	2.1E-14	9.9E-02	4.5E-01	6.7E-01	1.0E-16	1.0E-16
G1	1.1E-04	1.6E-14	9.1E-01	7.7E-01	1.0E-16	1.0E-16
Total group	1.2E-16	1.3E-15	8.3E-01	6.1E-01	1.0E-16	1.0E-15

^1 ^-- P values were calculated using Bartlett's bootstrap test. ^2 ^-- averaged P values were calculated using Box's M test (see description of procedures in Materials and methods section). U -- Uppsala cohort; S -- Stockholm cohort.

Bartlett test in Uppsala cohort showed that the tested correlation matrices were highly significant in all four different grades or using all patients data at significance level *α *= 1%. In the Stockholm cohort, G1 and G3 subgroups were highly significant at the same significant level. G3-like subgroup was close to the border line and only G1-like was not significant (Table 1). All NG matrices in both cohorts produced insignificant Bartlett P values and are not further considered as candidates for the members of *TNFAIP1/POLDIP2 *SFGM.

Next, we applied Box's M test to the comparison of two correlation matrices at *α *= 1%. The test revealed highly significant differences in almost all pairs of SFGM matrices and NG matrices (except for the Stockholm G3-like subgroup). Taken together, the statistical analysis clearly supports the existence of the five-gene SFGM. On the other hand, it strongly excludes the six other 'neighbouring' genes as members of this SFGM. We also utilized Box's M test to determine if there are any differences among SFGM matrices for each cancer grade in both cohorts. We observed that the SFGM showed a significant strengthening of its co-regulatory profile (from the first group to the second group in each pair, correspondingly) in the following group pairs: G1 and G3-like (Uppsala cohort, p = 8.08E-03; Stockholm cohort, p = 9.21E-07); G1-like and G3 (Stockholm cohort, p = 3.46E-004); G1 and G3 (Stockholm cohort, p = 2.62E-04); G1-like and G3-like (Stockholm cohort, p = 3.64E-08).

We suggest three possible mechanisms for the observed co-regulatory pattern of the *TNFAIP1/POLDIP2 *SFGM: an amplification mechanism (recurrent amplification), - if the modules are located in an amplified region on 17q involved in the process of breast cancer development; a chromatin remodeling/activation mechanism (for example, histone modification); and a transcription activation mechanism (for example, common regulatory transcription factors).

### Survival analysis of SFGM genes and their closest neighbours in breast cancer patients

We applied our survival analysis algorithm for the genes of SFGM and NG matrices. Four members (unique genes) of TNFAIP1/POLDIP2 SFGM are significant at *α *= 5% according to Wald P values, whereas no neighbouring genes satisfied this criterion. To minimize Type I error rate (false positives) we applied False Discovery Rate (FDR) correction to the P values using the classic FDR of Benjamini and Hochberg [27], extended for positive dependent data [28]. Typically, positive dependence exists if the variance covariance matrix of the six probes we study contains only positive entries, which is true in our case. At significance level *α *= 5%, the Uppsala and Stockholm cohort FDR corrected P values were estimated as  = 4.2*E *- 03 and  = 5.1*E *- 03, respectively. Table 2 indicates the Wald and FDR significant probes of our set. Notice that after FDR correction our set still contains highly significant genes in both cohorts. It is important that all four genes belong to the *TNFAIP1/POLDIP2 *SFGM and none belongs to the group including the six "neighbour genes". Interestingly, TMEM97 was survival significant in both cohorts, and it was shown previously to play a role in primary and metastatic colorectal cancers [29]. We also applied survival analysis and 2D data-driven grouping to identify survival significant probe pairs among the probes of our prospective cluster. First, we estimated the Wald P values and then used the FDR correction as before. The FDR corrected P values in Uppsala and Stockholm cohorts were  = 8.5*E *- 03 and  = 4.9*E *- 03. We kept the survival significant gene pairs which were common in the two cohorts. Table 3 shows our results (11 non-redundant survival significant gene pairs).

**Table 2:** Individual genes selected among the *TNFAIP1/POLDIP2 *SFGM and 6 "neighbouring" genes which proved to be survival significant in at least one cohort (P value ≤ 0.05).

Affymetrix U133 (A&B) probeset	Gene Symbol	Uppsala cohort(individual)		Stockholm P value (individual)	
		
		Wald statistic P value	FDR corrected P value = 4.2E-03	Wald statistic P value	FDR corrected P value = 5.1E-03
B.222425_s_at	POLDIP2	1.5E-05	Significant	2.4E-02	Not significant
A.210312_s_at	IFT20	4.1E-04	Significant	2.4E-02	Not significant
A.212282_at	TMEM97	1.1E-03	Significant	3.0E-03	Significant
B.225375_at	TMEM199	2.2E-02	Not significant	7.2E-04	Significant

The Uppsala cohort P value correction  = 4.2*E *- 03; the Stockholm cohort P value correction  = 5.1*E *- 03.

**Table 3:** Selected non-redundant survival-significant gene pairs identified in both cohorts of breast cancer patients

Affymetrix U133 (A&B) probeset	Gene Symbol	P value (individual)		Affyprobeset*	GS*	P value(individual)		P value(gene pair)	
					
		U	S			U	S	U	S
***222425_s_at***	***POLDIP2***	***1.50E-05***	***0.024***	***A.201207_at***	***TNFAIP1***	***0.00011***	***0.081***	***3.10E-07***	***0.00046***
***201208_s_at***	***TNFAIP1***	***0.022***	***0.11***	***A.214283_at***	***TMEM97***	***0.074***	***0.081***	***0.00022***	***0.0029***
***213259_s_at***	***SARM1***	***0.011***	***0.074***	***B.225375_at***	***TMEM199***	***0.022***	***0.00072***	***0.00085***	***2.90E-05***
204534_at	VTN	0.024	0.11	A.210312_s_at	IFT20	0.00041	0.024	0.00021	0.00052
204534_at	VTN	0.024	0.11	A.212279_at	TMEM97	0.0042	0.0035	1.00E-04	0.00062
210312_s_at	IFT20	0.00041	0.024	A.212281_s_at	TMEM97	0.0028	0.0051	1.60E-05	0.0036
213259_s_at	SARM1	0.011	0.074	A.212281_s_at	TMEM97	0.0028	0.0051	0.00039	0.004
217806_s_at	POLDIP2	4.30E-05	0.12	A.212281_s_at	TMEM97	0.0028	0.0051	2.60E-05	0.0036
225375_at	TMEM199	0.022	0.00072	A.201207_at	TNFAIP1	0.00011	0.081	9.30E-05	0.00021
225375_at	TMEM199	0.022	0.00072	A.212279_at	TMEM97	0.0042	0.0035	2.00E-04	0.00032

Bold italics indicates gene pairs where the P values for a gene pair are at least ten times lower than that for either of the individual gene's of the pair for both the Uppsala and Stockholm cohorts. U, Uppsala cohort; S, Stockholm cohort.

**Figure 4:**
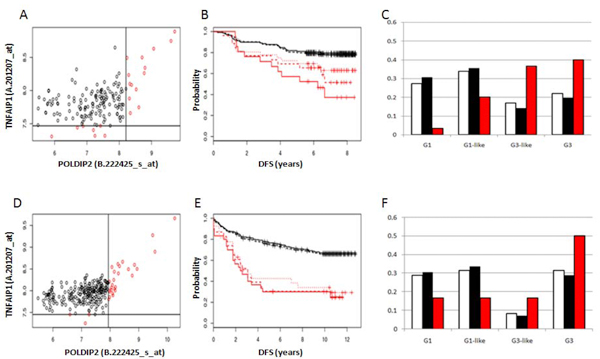
**Survival analysis for the TNFAIP1/POLDIP2 gene pair in breast cancer patients**. **A**, **B **and **C **-- plots and histogram for the Stockholm cohort; **D**, **E **and **F **- plots and histogram for the Uppsala cohort. Black indicates the low-risk prognosis group, red indicates the high risk prognosis group. **A **and **D **- correlation of gene expression and optimal partition of expression domains and patients grouping. The horizontal lines are the cut-offs of 2D data-driven grouping. **B **and **E **- Kaplan-Meier survival curves for *TNFAIP1 *and *POLDIP2 *when analyzed separately (*TNFAIP1 *- dotted line; *POLDIP2 *-- dashed line) as well as together (solid line). **C **and **F **-- separation of breast cancer patients based on expression data of the *TNFAIP1/POLDIP2 *gene pair in different grades [15]: Y axis -- frequency of patients in different groups; X axis -- patient distribution in different breast cancer grades (white column - total group; black column -- low-risk prognosis group; red column -- high risk prognosis group).

Among the seven unique genes that compose the eleven significant gene pairs (Table 3) we observed all five genes of the *TNFAIP1/POLDIP2 *SFGM and two (*SARM *and *VTN*) belonging to the 'neighbour's' gene group. Three of the eleven selected gene pairs (in bold italics in Table 3) demonstrated an effect of synergy, with the Wald P values for this more than ten times lower than the P values calculated for the individual genes of the pairs. - For example, the *TNFAIP1/POLDIP2 *(Figure 4), *TNFAIP1/TMEM97 *and *SARM1/TMEM199 *(Additional file 2, Figures S1 and S2) gene pairs revealed a synergistic effect with regard to survival in the Stockholm cohort. Figures 4, S1 and S2 show clearly that synergy improved patients grouping.

The gene pair *TNFAIP1/POLDIP2 *is a convergent SAGP in the middle of the *TNFAIP1/POLDIP2 *SFGM. Therefore, our survival analysis of the *TNFAIP1/POLDIP2 *SFGM and its "neighbouring genes" has revealed individual survival significant genes as well as significant gene pairs suggesting that the *TNFAIP1/POLDIP2 *SFGM is important for breast cancer prognosis.

### Expression of gene members of the *TNFAIP1/POLDIP2 *structural-functional gene module strongly correlates with DNA copy number

Previous studies of HER2-amplified tumors have demonstrated that the smallest region of amplification (SRA) involving HER2 spans 280 kb and contains a number of genes in addition to HER2 that have elevated levels of expression [32][33]. A comprehensive genomic study of *HER2 *(*ERBB2*) amplicon by Arriola *et al. *[30] revealed 21 additional smallest regions of amplification (SRAs) scattered throughout the genome. In our study of the data from [30] we found that the *TNFAIP1/POLDIP2 *SFGM is located inside of one of these 21 SRAs on 17q11.2 (genomic coordinates: 22, 766. 90 to 25, 931.27 kb [22]). We call this the *17q11.2 SRA *to distinguish it from the *TNFAIP1/POLDIP2 *SFGM. Correspondingly, the smallest region of amplification that includes the ERBB2 core region (CR; see below) as well as many other neighboring genes we call the *17q12 SRA *(genomic coordinates: 34, 730.32 to 35 476.80 kb) [30]).

**Figure 5:**
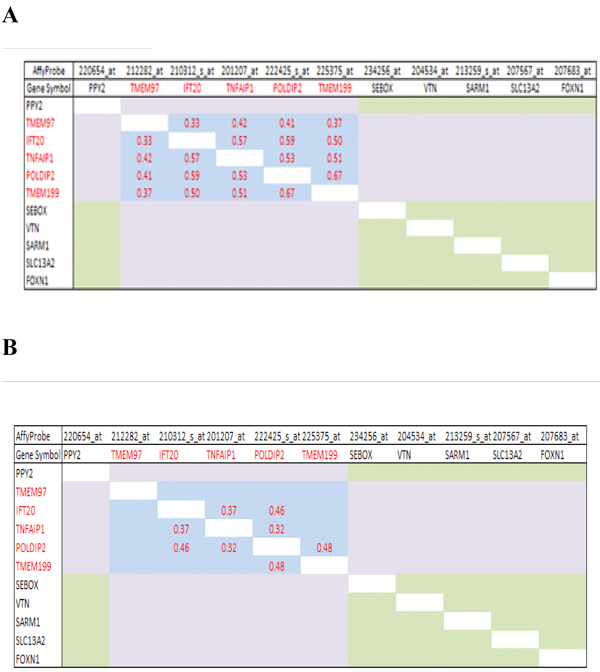
**Correlation matrices analysis of *TNFAIP1/POLDIP2* SFGM in 38 breast cancer cell lines (see materials and methods)**. Due to the small sample size (38 cell lines) Kendall-Tau correlation coefficients were calculated. Only significant correlation coefficients (*α *= 1%) are shown. **A. **Correlation matrix of the *TNFAIP1/POLDIP2 *SFGM produced by using original expression values (Additional file 3A). **B. **Correlation matrix of the *TNFAIP1/POLDIP2 *SFGM produced by using expression values normalized by DNA copy number (Additional file 3A).

In order to elucidate whether the mRNA expression levels of members of the *TNFAIP1/POLDIP2 *SFGM correlate with the DNA copy number of the corresponding region of the 17q11.2 SRA, we estimated Kendall-Tau correlation coefficients between DNA copy number values for selected SNPs and microarray expression data for the genes of the *TNFAIP1/POLDIP2 *SFGM as well as their neighbors. For this purpose we used high-resolution SNP microarray profiling together with microarray gene expression data (see Materials and methods) for 38 breast cancer cell lines for which both sources were available [12] (Additional file 3). Correlation matrix analysis with these 38 cell lines (Figure 5A) confirmed the clear co-regulatory pattern of the *TNFAIP1/POLDIP2 *SFGM, which was primarily identified in two breast cancer cohorts.

For the analysis of the *TNFAIP1/POLDIP2 *SFGM we selected four SNP markers covering the genomic region between 23, 333.55 and 24, 116.08 kb on the 17q11.2 SRA; the region of the *TNFAIP1/POLDIP2 *SFGM and neighboring genes covers the region between 23, 598.60 kb (the start of the *PPY2 *gene in the UCSC browser) and 23, 889.23 kb (the end of the *FOXN1 *gene). The results of the correlation analysis are presented in Table 4 that shows significant correlations of expression with DNA copy number for all genes of the *TNFAIP1/POLDIP2 *SFGM. Hence, amplification of the 17q11.2 SRA can be an important driver of expression for the genes of the *TNFAIP1/POLDIP2 *SFGM in breast cancer.

**Table 4:** Correlation analysis of DNA copy number and microarray expression data for the *TNFAIP1/POLDIP2 *SFGM and *ERBB2 *CR.

Affy probesets*	Gene Symbol	SNPs for 17q11.2 SRA				SNPs for 17q12 SRA						
			
		rs4239211	rs10512429	rs7207976	rs10512430	rs602688	rs632202	rs620686	rs10491129	rs10491128	rs2517956	rs9303277
220654_at	PPY2											
**212282_at**	**TMEM97**	**0.29**										
**210312_s_at**	**IFT20**	**0.41**	**0.39**	**0.39**	**0.39**							
**201207_at**	**TNFAIP1**	**0.40**	**0.33**	**0.33**	**0.33**							
**222425_s_at**	**POLDIP2**	**0.45**	**0.39**	**0.39**	**0.39**				0.29		0.34	0.34
**225375_at**	**TMEM199**	**0.48**	**0.41**	**0.41**	**0.41**							
234256_at	SEBOX											
204534_at	VTN											
213259_s_at	SARM1											
207567_at	SLC13A2											
207683_at	FOXN1											

**200029_at**	**RPL19**					**0.43**	**0.43**	**0.43**	**0.42**	**0.35**		
228888_at	STAC2										0.35	0.35
**239224_at**	**FBXL20**					**0.54**	**0.54**	**0.54**	**0.50**	**0.48**	**0.39**	**0.39**
**203497_at**	**PPARBP**					**0.58**	**0.58**	**0.58**	**0.63**	**0.68**	**0.54**	**0.54**
**213557_at**	**CRKRS**					**0.45**	**0.45**	**0.45**	**0.50**	**0.57**	**0.48**	**0.48**
210271_at	NEUROD2											
**225165_at**	PPP1R1B**											
**202991_at**	**STARD3**								**0.32**	**0.39**	**0.50**	**0.50**
**205766_at**	TCAP**											
**206793_at**	**PNMT**											
**221811_at**	**PERLD1**									**0.32**	**0.39**	**0.39**
**216836_s_at**	**ERBB2**										**0.42**	**0.42**
**224447_s_at**	**C17orf37**									**0.30**	**0.50**	**0.50**
**210761_s_at**	**GRB7**									**0.32**	**0.49**	**0.49**
221092_at	IKZF3											
231442_at	ZPBP2											
**219233_s_at**	**GSDML**					**0.39**	**0.39**	**0.39**	**0.36**	**0.42**	**0.41**	**0.41**

Only Kendal-Tau correlation coefficients with *α *= 1% are shown. Bold font indicates genes shown to be involved in the co-regulatory pattern of the *TNFAIP1/POLDIP2 *SFGM and *ERBB2 *amplicon. *Affymetrix U133A&B probesets, **PPP1R1B and TCAP - genes of the *ERBB2 *amplicon that have been excluded previously from the *ERBB2 *CR due to their weak expression and nonsignificant correlation with DNA copy number [32,33].

### Expression of gene-members of the *TNFAIP1/POLDIP2 *SFGM strongly correlates with expression of gene-members of the ERBB2 core region

The *TNFAIP1/POLDIP2 *SFGM is located on 17q11.2 centromeric to the region of the *ERBB2 *locus on the 17q12 cytoband. The *ERBB2 *locus has been extensively studied and has been proposed to be one of the most important loci in breast cancer [31]. It is widely accepted, that the most common mechanism for ERBB2 activation in breast cancer is gene amplification [32-34]. It is also well established that the amplified DNA segment (amplicon) in breast cancer is often rather large and typically covers many genes [35].

In our analysis we found that the genes composing the *TNFAIP1/POLDIP2 *SFGM are located in a much wider region of the 17q11.2 SRA (see above for definition) [30]. According to Arriola *et al. *[30], this region (together with 20 other regions in the human genome) is associated with *HER2 (ERBB2)- *and *HER2/TOP2A*-amplified breast tumors. In another study it was suggested that predominantly luminal and *HER2 (ERBB2) *cancers display a characteristic 'firestorm/amplifier' genomic pattern [36], that is, when coamplification of many regions in the genome is observed as a common phenomenon.

Previous studies on the ERBB2 amplicon have utilized several different approaches. One of these was based on detection of a correlation between DNA copy number and mRNA expression [32,33]. This approach was successfully used for characterization of the *ERBB2 *amplicon and determination of its smallest minimal region of amplification, the core region of the *ERBB2 *amplicon (the *ERBB2 *CR) which is a 280 kb long [32,33]. The analysis showed that the *ERBB2 *CR includes the following genes: *ERBB2, GRB2, STARD3, PP1R1B, PNMT, NEUROD2, TCAP, ZNFN1A3, PERLD1 *and *C17orf37*. Real-time RT-PCR confirmed the correlation between amplification and expression levels [32] for genes comprising the *ERBB2 *CR. Finally, the *ERBB2 *CR was suggested to include the genes *ERBB2, GRB2, STARD3, PNMT, PERLD1 *and *C17orf37 *[32,33]. Therefore, the borders of the *ERBB2 *CR could be defined by the *STARD3* gene centromerically and the *GRB7* gene telomerically.

In our study, we performed DNA copy number analysis of the *ERBB2 *CR together with several flanking genes; for this purpose we used seven SNP markers covering the region from 34, 693.02 to 35, 229. 99 kb on the 17q12 SRA [12,30]. For the Affymetrix microarray expression analysis the data for the genes of the *ERBB2 *CR as well as their closest neighbor genes (the region between 34, 610.06 (the start of *RPL19*) and 35, 337. 38 kb (the start of *ORMDL3*)) were utilized. Correlation analysis of the expression profile with DNA copy number was performed exactly as for the *TNFAIP1/POLDIP2 *SFGM (see the previous section) [12]. The results of the correlation analysis of the *ERBB2 *CR are presented in Table 4. As expected, the expression-copy number correlation pattern for the genes of the *ERBB2 *CR in our analysis demonstrated good consistency with the data of Kauraniemi *et al. *[32,33].

Another approach originally applied to budding yeast [37] and *Drosophila *[38] included *searching (or prediction) *for groups of neighboring genes that showed correlated expression profiles. The same idea was utilized for the human genome [39]. The transcription correlation score was calculated for each gene in the genome. The score was calculated as the sum of the Spearman rank order correlation values in the tumor samples between the RNA levels of the gene of interest and the RNA levels of each of the physically nearest 2n genes (n centromeric genes and n telomeric genes). Specifically, our analysis of supplementary material for the *ERBB2 *amplicon in [39] showed that this second approach confirmed the data on the basic members of the *ERBB2 *amplicon and, therefore, showed good consistency with the first approach [39].

In our correlation analysis we applied a similar idea as in [39] but used a different *computational apparatus *(see Materials and methods) and performed more detailed characterization of genomic regions of interest. In order to validate the reliability of our approach, originally applied to the characterization of the *TNFAIP1/POLDIP2 *SFGM, we performed a similar correlation analysis of matrices for the region of the *ERBB2 *amplicon which has been well-characterized previously.

**Figure 6:**
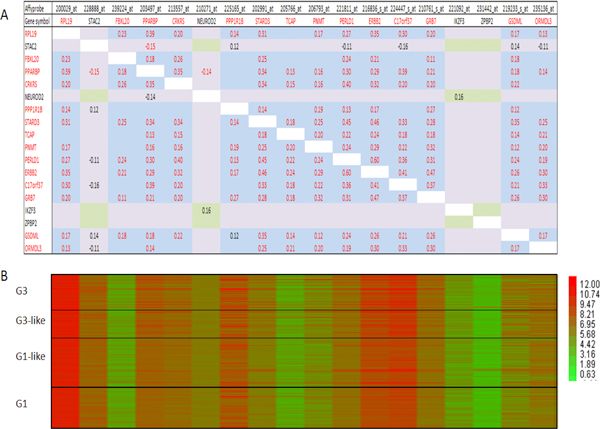
**Correlation matrix for *ERBB2 *amplicon (Uppsala cohort)**. **A **-- correlation matrix (Pearson, *α *= 1%); **B **- heat map analysis in different breast cancer grades.

**Figure 7:**
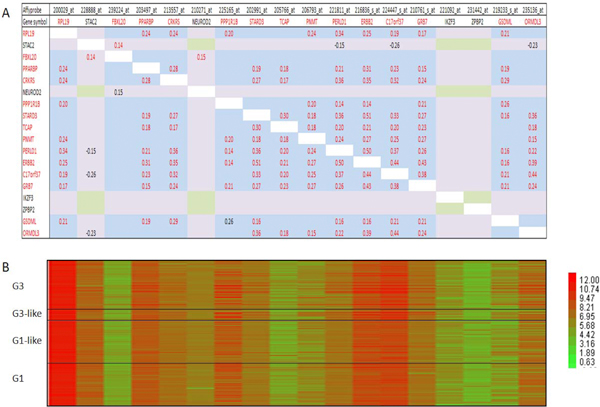
**Correlation matrix for *ERBB2 *amplicon (Stockholm cohort)**. **A **-- correlation matrix (Pearson, *α *= 1%); **B **- heat map analysis in different breast cancer grades.

We produced correlation matrices that included 6 validated genes of the *ERBB2 *CR (see above) and 12 neighboring genes based on data from the Uppsala and Stockholm cohorts. Genes in a matrix were placed one by one in the order of their chromosome localization (from RPL19 centromerically to ORMDL3 telomerically; Figures 6 and 7). Interestingly, we reproducibly (in both cohorts) observed a clear co-regulatory pattern (as we did for the *TNFAIP1/POLDIP2 *SFGM) of a transcriptional module that included not only the genes that were validated as members of the *ERBB2 *CR (see above and [33]), but also - the genes *TCAP *and *PPP1R1B *(Figures 6 and 7, Z-value correlation, *α *= 1%). The neighboring genes *NEUROD2, IKZF3 (ZNFN1A3) *and *ZPBP2 *were 'dropped' from the module due to a lack of significant correlations with any member of the module and matrix.

Independently, for the 38 breast cancer cell lines for which both expression and DNA copy number data were available [12] (and which were used in our DNA copy number analysis shown in Table 4) the correlation matrix for the ERBB2 CR and its neighboring genes was very similar (Additional file 4) to those produced for the breast cancer cohorts (Figures 6 and 7).

Previously, Kauraniemi *et al. *[33] excluded *TCAP *and *PPP1R1B *as well as *NEUROD2 *and *IKZF3 (ZNFN1A3) *from the *ERBB2 *CR based on their weak or absent expression and lack of correlation with copy number. Therefore, the results of our *ERBB2 *CR matrix correlation analysis demonstrate good consistency with the data of Kauraniemi *et al. *[32,33], who used a different approach. Similarly, in the study of Reyal *et al. *[39] only genes *NEUROD2, PPP1R1B, IKZF3 *and *ZPBP *were absent from the list of genes for which the transcription correlation score was above the threshold for the transcription correlation map of 130 invasive ductal carcinomas.

Our correlation analysis of the *ERBB2 *CR and neighboring genes in the chosen genomic window is not only in a good agreement with previous studies based on different approaches, but also adds new information that could be methodologically important. In this context, we suggest that the correlation matrix analysis we have applied in the present work could be a new independent tool for studying of amplified and/or co-regulated genomic regions in cancer.

**Figure 8:**
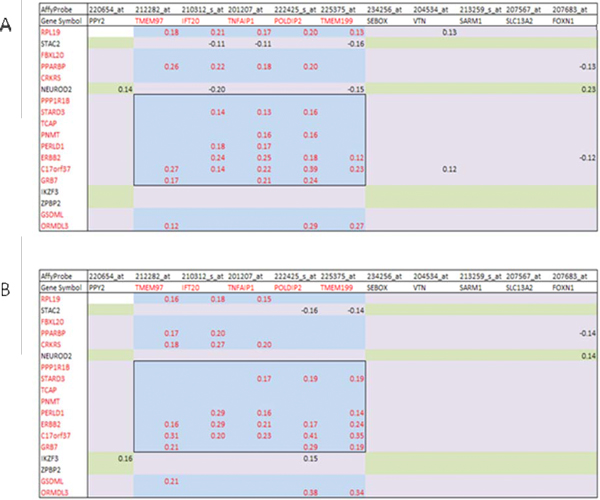
**Correlation tables between the genes of the *TNFAIP1/POLDIP2 *SFGM and its 'neighbours' and the *ERBB2 *CR and its 'neighbours' in breast cancer patients**. The central selected area of the matrix represents significant correlations (Pearson, *α *= 1%) between the *TNFAIP1/POLDIP2 *SFGM and the *ERBB2 *CR. **A **-- Uppsala cohort, **B **-- Stockholm cohort.

Due to the previously documented fact of co-amplification of broad genomic regions of the 17q11.2 and 17q12 SRAs [30] at the DNA level, we proposed that expression of the genes composing the *TNFAIP1/POLDIP2 *SFGM (located inside the 17q11.2 SRA) and genes composing the *ERBB2 *CR (located inside the 17q12 SRA) could also be correlated at the level of transcription. We produced correlation tables that included both the genes of the *TNFAIP1/POLDIP2 *SFGM and the ERBB2 CR and their neighboring genes in the Uppsala and Stockholm breast cancer cohorts. We found that the mRNA expression levels of all members of the *TNFAIP1/POLDIP2 *SFGM were significantly correlated with those of at least two or more members of the *ERBB2 *CR (Figure 8A, B). *ERBB2 *and *C17orf37 *were significantly correlated with almost all (except *TMEM97*) members of the *TNFAIP1/POLDIP2 *SFGM in both cohorts.

Similarly, in 38 breast cancer cell lines (Additional file 5), the expression of the *ERBB2 *gene was significantly correlated with the expression of all the 5 members of the *TNFAIP1/POLDIP2 *SFGM, although the total number of observed significant correlations was less than for the breast cancer patients (Figures 8A, B).

Therefore, the expression profiles of the genes of the *TNFAIP1/POLDIP2 *SFGM and the *ERBB2 *CR are correlated in breast cancer and this fact could probably be explained by co-amplification of their genomic regions. However, alternative mechanisms could be considered, including similar epigenetic modifications of chromatin as well as common upstream regulatory transcription factors.

### Genes of the *TNFAIP1/POLDIP2 *SFGM are co-regulated not only through changes in DNA copy number but also by transcription activation and chromatin remodelling

Figure 2B illustrates several findings that could indicate histone modification as a possible mechanism of the observed transcriptional co-regulatory pattern of the *TNFAIP1/POLDIP2 *SFGM genes. Custom tracks in the UCSC Genome Browser for trimethylated histones H3K4me3 and H3K27me3 (promyelocytic leukemia cells (HL60) [40], http://www.bcgsc.ca/data/histone-modification confirmed the transcriptional activation of the genes involved in the *TNFAIP1/POLDIP2 *SFGM. All three CpG-rich putative promoters in the *TNFAIP1/POLDIP2 *SFGM showed clear signal for H3K4me3 (a marker of transcriptionally active chromatin) as well as a lack of signal for H3K27me3 (a marker for inactive chromatin). Nevertheless, putative promoters for the 'neighbouring' genes *SEBOX*, *VTN*, and *SARM *did not show any signal of  H3K4me3. A similar situation is observed with the GIS Chip-PET track (embryonic stem cells hES3) of the UCSC Browser [41] (Figure 2B). A strong signal for H3K4me3 is observed in all three putative promoter regions of the *TNFAIP1/POLDIP2 *SFGM and only weak signal of H3K27me3 is detected for the *TMEM97 *and *TMEM199/POLDIP2 *putative promoters. Alternatively, the putative promoter for the *SEBOX *gene does not show any signal for both H3K4me3 and H3K27me3; the regulatory region of the *VTN *and *SARM1 *genes reveals moderate signal for H3K4me3 and H3K27me3 of the same intensity.

Additional custom tracks in the UCSC browser for STAT1 binding in HeLa S3 cells [42] clearly demonstrate the presence of two functional STAT1 binding sites in the *IFT20/TNFAIP1 *bidirectional promoter. Moreover, upon stimulation by interferon-gamma, the binding signal intensity for STAT1 increased at least seven-fold (Figure 2B). Recently, Liu *at al. *[43] reported that another transcription factor, Sp1, is directly (*in vivo*) associated with the *TNFAIP1 *promoter in HeLa cells. Therefore, the *TNFAIP1/POLDIP2 *SFGM is also potentially regulated by STAT1 and Sp1 as well as stimulated by interferon-gamma in breast cancer cells. Finally, direct and independent evidence of the *TNFAIP1/POLDIP2 *SFGM activation in breast cancer cells comes from the custom track for RNA polymerase II binding in the MCF7 breast cancer cell line (Figure 2B, black arrows) [44].

Results of our additional experiment are presented in Figure 5B. We produced the correlation matrix of the *TNFAIP1/POLDIP2 *SFGM based not on the gene expression values (for 38 cell lines [12]; Figure 5A), but on their ratios to DNA copy number values (normalized expression values). Expression data originally extracted from [12] as well as data normalized to DNA copy number are presented in Additional file 3. Because the values for all four SNP markers utilized in the analysis of the 38 breast cancer cell lines were identical (Additional file 3), we were able to perform normalization using values for any of them. The produced matrix revealed the typical co-regulatory pattern again, although with fewer mutual correlations.

Therefore, we have clearly demonstrated that not only recurrent amplification, but also chromatin remodeling and/or transcription activation is important for the establishment and maintenance of the co-regulatory pattern of the *TNFAIP1/POLDIP2 *SFGM. Moreover, we suggest that the co-regulatory pattern of the five member genes of the *TNFAIP1/POLDIP2 *SFGM could originally be established as the result of epigenetic modifications and/or transcriptional activation rather than by recurrent amplification in breast cancer cells. Theoretically, the latter mechanism could serve as an 'accelerator' of an already established preexisting co-regulatory pattern. In this context, the role of the CSAGA in the *TNFAIP1/POLDIP2 *SFGM deserves special attention and comprehensive experimental study.

## Discussion

### A method for the statistical identification of co-regulated genes organized in complex genome architectures

In the present study, we have developed a new computational method for the statistical identification of co-regulated genes organized in complex genome architectures including more than one SAGP. Our approach is based on: (i) concordant analysis and selection of expressed SA genes; (ii) identification of the boundaries of a genomic region encompassing genes with similar co-expression patterns; (iii) validation of the expression pattern using independent patient cohorts; (iv) evaluation of the clinical significance of expressed genes that belong to the identified genome region; and (v) identification of the synergy of the genes in the context of disease aggressiveness and disease relapse.

### *TNFAIP1/POLDIP2 *is an essential structural-functional module in the human genome

We analyzed the *TNFAIP1/POLDIP2 *CSAGA on 17q11.2 in two breast cancer cohorts. The *TNFAIP1/POLDIP2 *CSAGA is composed of five genes: *TMEM97, IFT20, TNFAIP1, POLDIP2 *and *TMEM199*. The gene pairs *TMEM97/IFT20*, *TNFAIP1/POLDIP2 *and *IFT20/TNFAIP1 *produce sense-antisense transcripts; the gene pairs *IFT20/TNFAIP1 *and *POLDIP2/TMEM199 *share corresponding bi-directional promoter regions. This complex genomic region exhibits a well-organized transcription apparatus: 3 CpG islands; two experimentally validated (*STAT1* and *Sp1*) and several putative transcription factor binding sites in canonical promoter regions - *GATA1*, *TAXCREB*, *CREBP1*, *CREB *and *SREBP1 *Transfac 7.0); strong signals for RNA polymerase II binding (Figure 2B); and probable open chromatin regions (H3K4met3(+) and H3K27met3(-)) (Figure 2B). The *TNFAIP1/POLDIP2 *CSAGA region could produce a large diversity of alternative splice variants of the genes it encompasses (USCS Genome Browser, AceView Gene Models with Alternative Splicing). Our analysis of correlation matrices revealed a phenomenon whereby genes structurally organized in the genome in the CSAGA demonstrate a reproducible co-regulatory pattern in breast cancer cells (Figure 3). We termed the *TNFAIP1/POLDIP2 *CSAGA the TNFAIP1/POLDIP2 SFGM.

### Concordant regulation in the *TNFAIP1/POLDIP2 *CSAGA

We did not observe any significant negative correlations (discordant regulation) in the *TNFAIP1/POLDIP2 *SFGM in agreement with several previous reports of frequent concordant regulation of sense-antisense pairs [45-47].

Correlation analysis of the *TNFAIP1/POLDIP2 *SFGM in four grades of breast cancer (G1, G1-like, G3-like and G3) revealed a strengthening of the correlations between the genes of the *TNFAIP1/POLDIP2 *SFGM. Survival analysis of individual genes as well as of gene pairs from the *TNFAIP1/POLDIP2 *SFGM and its neighbors was also performed. Only the genes of the *TNFAIP1/POLDIP2 *SFGM proved to be survival significant in at least one of the two cohorts analyzed (Table 2). Among 11 genes analyzed, 10 survival-significant gene pairs have been identified and all the genes of the *TNFAIP1/POLDIP2 *SFGM were involved in these pairs. Each of the 11 pairs contained at least 1 gene from the SFGM. Moreover, three top level survival-significant gene pairs demonstrated a synergistic effect with regard to the prognosis of breast cancer disease relapse when compared with individual genes (Table 3). This finding indicates the importance of this module in breast cancer progression and prognosis.

### Protein interaction sub-network

Our analysis of the literature on the members of the *TNFAIP1/POLDIP2 *SFGM confirmed a previous suggestion regarding its functional integrity and its possible importance in cancers.

Liu *et al. *[23] reported on the physical interaction of the *POLDIP2 *protein with the *p50 *subunit of DNA polymerase delta and *PCNA*. *PCNA *has been called the 'ringmaster of the genome', because it has been shown to actively participate in a number of the molecular pathways responsible for the life and death of the mammalian cell [48]. It marker to evaluate cell proliferation and prognosis when combined with other breast cancer markers, such as estrogen receptor, progesterone receptor and *ERBB2 *[49-51].

*TNFAIP1 *belongs to *KCTD *family of the proteins containing T1 domain capable of regulation of the voltage-gated potassium channels. It was shown that rat *TNFAIP1 *is highly homologous to polymerase delta-interacting protein (*PDIP1*) as well as to KCTD10 and all three proteins can directly interact with *PCNA*. In the rat, *PDIP1*, *TNFAIP1 *and *KCTD10 *can stimulate DNA polymerase delta activity *in vitro *in *PCNA*-dependent way [25,52]. Of note, down regulation of *KCTD10 *can inhibit cell proliferation in carcinoma A549 cells [53]. Direct indications of involvement of TNFAIP1 in apoptosis and carcinogenesis include the following facts: - CK2-mediated phosphorylation of *TNFAIP1 *in HeLa cells affects its sub-cellular localization and interaction with *PCNA *[54]; RhoB induces apoptosis by direct interaction with *TNFAIP1 *in HeLa cells [55].

*TMEM97 *cytoplasmic expression was shown to be positively correlated to expression of *PCNA*; this gene is considered a prognostic factor in the metastasis of colorectal cancer [29]. Another important fact is that in UV-irradiated human cells, *PCNA *foci demonstrate striking colocalization with phosphorylated breast cancer susceptibility protein *BRCA1*[56]. Both *PCNA *and *BRCA1 *are required for postreplication repair [57]. Therefore, at least three members of the *TNFAIP1/POLDIP2 *module could be functionally associated in the same *PCNA *complex.

Two interesting recent publications support the idea about the involvement of the *TNFAIP1/POLDIP2 *module in the cell cycle and cell proliferation: POLDIP2 was shown to be associated with spindle organization and aberrant chromosome segregation [58]; and tissue-specific deletion of floxed *IFT20 *in the mouse kidney causes mis-orientation of the mitotic spindle in collecting duct cells, prevents cilia formation and promotes rapid postnatal cystic expansion of the kidney [59].

Interesting pleiotropic effects of *POLDIP2 *also include interaction with cell-cell adhesion receptor *CEACAM1 *[60] and involvement in transcription and metabolism of mitochondrial DNA [61].

### Co-regulatory pattern of the *TNFAIP1/POLDIP2 *SFGM with the *ERBB2 *amplicon

It is important to note that the *TNFAIP1/POLDIP2 *module is located outside of the well-known *ERBB2 *amplicon on 17q12, over-representation of which in the genome is often associated with the occurrence of the *ERBB2*-positive breast cancer subtype. In the present work, we demonstrated reproducible correlations of the *TNFAIP1/POLDIP2 *SFGM with the 'core region' of the ERBB2 amplicon (Figure 8). This finding is in good agreement with data from a recent report on *HER2 *(*ERBB2*) co-amplified regions in breast cancer patients and cell lines [30]. In fact the *TNFAIP1/POLDIP2 *SFGM is located inside the smallest region of recurrent amplification on 17q11.2 and expression of its members strongly correlates with DNA copy number (see the Results section). Significant correlations between members of both modules could be explained, at least partially, by a co-amplification mechanism. Nevertheless, the correlation of the expression profiles of these modules would not imply a direct association with similar breast cancer subtype.

It is well established that overexpression of the *ERBB2 *amplicon is predominantly associated with the *ERBB2 *breast cancer subtype [13,14]. Preliminary data obtained in our pilot study (not shown) indicate that the *TNFAIP1/POLDIP2 *SFGM demonstrates stronger correlation pattern not with the *ERBB2 *breast cancer subtype but rather with luminal A and B subtypes.

Therefore, we suggest that the *TNFAIP1/POLDIP2 *SFGM could be used potentially as a new integrative indicator in breast cancer diagnosis, prognosis and treatment monitoring. This issue requires comprehensive study and will be addressed in future publications.

Taken together, our analysis suggests that the *TNFAIP1/POLDIP2 *SFGM is composed of genes that are not only closely organized in a complex genomic architecture and co-regulated at the transcription level, but also could be involved in essential common biochemical pathways as well as protein-protein and protein-DNA interactions forming molecular complexes important for many cellular processes, including cell division, proliferation, apoptosis, intracellular transport and cell binding. Such diverse structural and functional properties suggest the biological importance and clinical significance of the *TNFAIP1/POLDIP2 *CSAGA.

## Conclusion

We conclude that the methods of computational identification of *novel structural and functional gene modules *and the grouping of clinically heterogeneous (cancer) patients based on the expression patterns of genes of these modules could provide broad perspectives for the development of computational systems biology strategies for understanding the genetics and pathobiology of many complex genetic diseases.

Due to concordant regulation of the genes in such modules, one could target just the antisense transcript(s), resulting in reduction of sense mRNA transcripts, or also the adjacent genes of the module, thereby achieving additive and even synergistic reduction of expression of a specific group of neighboring genes [62]. Pharmacological strategies aimed at either stimulation or suppression of expression of a specific group of genes that are influenced by natural SA regulation could also be developed. A discovery of biologically meaningful and clinically significant CSAGAs, instead of the conventional finding of 'gene signatures', might be more promising in the context of the appropriate translation of microarray analyses into clinical practice and the identification of new drug development strategies.

## List of abbreviations used

SA: sense-antisense; SAGP: Sense-antisense gene pair; CSAGA: complex sense-antisense gene architecture; GEO: Gene Expression Omnibus; *SFGM*: *structural-functional gene module*; *FDR*: *False Discovery Rate*; *CR*: *core region*

## Competing interests

The authors declare that they have no competing interests.

## Authors' contributions

V.A.K. initiated the study, developed general conception and provided interpretation of the results and leaded the project. O.V.G. designed and implemented the study framework in order to apply bioinformatics tools and statistical approaches for biological interpretation of the obtained results. E.M. provided statistical analysis, computer simulations, and programming. All the authors were actively involved in writing of the draft and preparing of final version of the manuscript.

## Supplementary Material

Additional file 1 **P-values calculated by Kolmogorov-Smirnov test of Normality (*α *= 1%)**. Description: file contains two tables with P-values of Normality for the five genes of *TNFAIP1/POLDIP2 *SFGM and six their neighbours and two tables with P-values of Normality for 17 genes of the *ERBB2 *amplicon. Click here for file

Additional file 2 **Survival analysis for the *TMEM97/TNFAIP1 *(Figure S1) and *TMEM199/SARM1 *(Figure S2) gene pairs**. Description: file contains patients grouping and Kaplan-Meier survival curves for the *TMEM97/TNFAIP1 *and *TMEM199/SARM1 *gene pairs in the Uppsala and Stockholm breast cancer cohorts. Click here for file

Additional file 3 **Expression data for 38 breast cancer cell lines either non-normalized or normalized to DNA copy number**. File contains the original expression values for 38 breast cancer cell lines extracted from Hu *et al. *[12] as well as the expression values normalized to DNA copy number. Click here for file

Additional file 4 **Correlation matrix of the genes involved in the *ERBB2 *CR in breast cancer cell lines**. File represents correlation matrix analysis of the genes involved in the ERBB2 CR as well as 11 'neighbouring' genes in a sample of 38 breast cancer cell lines (Kendall-Tau, *α *= 1%). Click here for file

Additional file 5 **Correlation analysis between the genes of the *TNFAIP1/POLDIP2 *SFGM and the the *ERBB2 *CR in breast cancer cell lines**. File represents correlation analysis between the genes of the *TNFAIP1/POLDIP2 *SFGM and its 'neighbours' and the genes involved in the *ERBB2 *CR and its 'neighbour' genes in 38 breast cancer cell lines (Kendal-Tau, *α *= 1%). Click here for file
