# Supplementary material for: Complex sense-antisense architecture of TNFAIP1/POLDIP2 on 17q11.2 represents a novel transcriptional structural-functional gene module involved in breast cancer progression
Source: BMC Genomics. 2010 Feb 10;11(Suppl 1):S9. doi: 10.1186/1471-2164-11-S1-S9 (PMC2822537; doi:10.1186/1471-2164-11-S1-S9)
Supplement: Additional file 1 — P-values calculated by Kolmogorov-Smirnov test of Normality (α = 1%). Description: file contains two tables with P-values of Normality for the five genes of TNFAIP1/POLDIP2 SFGM and six their neighbours and two tables with P-values of Normality for 17 genes of the ERBB2 amplicon. [file 1471-2164-11-S1-S9-S1.pdf]

Supplementary Table S1a. P values of Kolmogorov-Smirnov test of Normality in the Stockholm cohort for 5 genes in SFGM group (SFGM matrix) and 6 "neighbouring" genes (NM – "neighbours" matrix). The test is calculated for all Stockholm cohort data and by breast cancer grade. P values higher than 0.01 indicate approximately Normally distributed data.

		ALL_DATA	GRADE_1	GRADE_1-LIKE	GRADE_3-LIKE	GRADE_3
212282_at	TMEM97	1.20E-01	3.46E-01	8.90E-01	7.98E-01	4.07E-01
210312_s_at	IFT20	6.05E-01	7.57E-01	6.23E-01	9.67E-01	2.88E-01
201207_at	TNFAIP1	1.75E-01	8.98E-01	8.73E-01	9.45E-01	5.74E-01
222425_s_at	POLDIP2	4.83E-01	6.40E-01	7.29E-01	6.18E-01	4.84E-01
225375_at	TMEM199	4.01E-01	3.37E-01	5.93E-01	8.58E-01	4.54E-01
234256_at	SEBOX	7.86E-02	3.67E-01	6.08E-01	9.59E-01	2.26E-01
204534_at	VTN	4.76E-03	3.99E-02	1.47E-01	6.78E-01	2.41E-01
213259_s_at	SARM1	1.89E-01	5.23E-01	7.49E-01	9.23E-01	7.85E-01
207567_at	SLC13A2	4.83E-02	3.14E-01	4.13E-01	9.96E-01	1.79E-01
207683_at	FOXN1	5.03E-01	8.95E-01	8.76E-01	6.70E-01	9.20E-01
220654_at	PPY2	1.88E-01	4.05E-01	3.71E-01	8.47E-01	8.32E-01

Supplementary Table S1b. P values of Kolmogorov-Smirnov test of Normality in the Uppsala cohort for 5 genes in SFGM group (SFGM matrix) and 6 "neighbouring" genes (NM – "neighbours" matrix). The test is calculated for all Stockholm cohort data and by breast cancer grade. P values higher than 0.01 indicate approximately Normally distributed data.

		ALL_DATA	GRADE_1	GRADE_1-LIKE	GRADE_3-LIKE	GRADE_3
212282_at	TMEM97	1.54E-04	7.27E-01	1.64E-02	4.66E-01	1.62E-01

210312_s_at	IFT20	7.47E-02	7.42E-01	5.29E-01	5.76E-01	6.22E-01
201207_at	TNFAIP1	8.05E-03	4.41E-01	9.85E-01	4.83E-01	3.26E-01
222425_s_at	POLDIP2	3.55E-02	2.99E-01	1.82E-01	6.01E-01	6.86E-01
225375_at	TMEM199	8.43E-02	1.65E-01	3.93E-01	6.61E-01	2.52E-01
234256_at	SEBOX	4.51E-03	4.46E-02	8.50E-01	2.05E-01	4.25E-01
204534_at	VTN	2.02E-04	4.23E-01	7.89E-03	3.59E-02	3.53E-01
213259_s_at	SARM1	2.85E-01	3.43E-01	9.16E-01	5.74E-01	8.62E-01
207567_at	SLC13A2	2.34E-02	2.41E-01	2.41E-02	2.69E-01	7.61E-01
207683_at	FOXN1	2.45E-01	4.57E-01	7.79E-01	2.77E-01	4.62E-01
220654_at	PPY2	3.64E-01	3.30E-01	6.57E-01	9.24E-01	8.64E-01

Supplementary Table S1c. P values of Kolmogorov-Smirnov test of Normality in Stockholm cohort for 17 genes of ERBB2 amplicon on 17q12. The test is calculated for all Stockholm cohort data and by breast cancer grade. P values higher than 0.01 indicate approximately Normally distributed data.

		ALL_DATA	GRADE_1	GRADE_1-LIKE	GRADE_3-LIKE	GRADE_3
200029_at	RPL19	1.43E-01	8.42E-01	1.58E-01	7.93E-01	7.01E-01
228888_at	STAC2	2.65E-01	2.76E-01	9.16E-01	9.65E-01	4.41E-01
239224_at	FBXL20	1.21E-01	9.62E-01	7.58E-01	3.98E-01	7.30E-01
203497_at	PPARBP	4.79E-03	3.14E-01	4.43E-01	8.62E-01	5.43E-02
213557_at	CRKRS	6.90E-03	4.90E-01	6.00E-01	9.33E-01	2.49E-01
210271_at	NEUROD2	1.60E-02	4.40E-03	9.04E-01	1.33E-01	9.87E-01
225165_at	PPP1R1B**	3.34E-01	7.86E-01	7.62E-01	2.84E-01	2.96E-01
202991_at	STARD3	1.01E-05	3.83E-02	5.25E-01	9.04E-01	3.53E-03
205766_at	TCAP**	2.86E-02	3.74E-01	2.16E-02	9.30E-01	8.62E-01
206793_at	PNMT	6.75E-02	3.70E-01	6.18E-01	9.79E-01	1.20E-01

221811_at	PERLD1	5.86E-05	2.55E-02	6.36E-01	7.69E-01	2.07E-01
216836_s_at	ERBB2	3.19E-03	4.78E-01	2.70E-01	8.67E-01	1.25E-01
224447_s_at	C17orf37	1.36E-04	4.83E-01	2.76E-01	3.22E-01	1.89E-01
210761_s_at	GRB7	2.98E-03	3.00E-01	7.04E-01	3.03E-01	2.41E-02
221092_at	IKZF3	7.68E-03	1.68E-01	1.27E-01	5.97E-01	2.93E-01
231442_at	ZPBP2	1.51E-01	3.02E-01	2.79E-01	3.78E-01	2.52E-01
219233_s_at	GSDML	3.28E-01	7.99E-01	5.03E-01	9.39E-01	7.32E-01

Supplementary Table S1d. P values of Kolmogorov-Smirnov test of Normality in the Uppsala cohort for 17 genes of the ERBB2 amplicon on 17q12. The test is calculated for all Stockholm cohort data and by breast cancer grade. P values higher than 0.01 indicate approximately Normally distributed data.

		ALL_DATA	GRADE_1	GRADE_1-LIKE	GRADE_3-LIKE	GRADE_3
200029_at	RPL19	2.20E-02	8.64E-01	6.06E-01	9.10E-01	3.62E-01
228888_at	STAC2	2.81E-01	5.73E-01	4.60E-01	8.45E-01	7.69E-01
239224_at	FBXL20	2.62E-02	3.84E-01	5.19E-02	1.05E-01	6.88E-03
203497_at	PPARBP	5.54E-12	3.89E-01	6.20E-03	2.52E-02	1.51E-02
213557_at	CRKRS	5.39E-08	9.06E-01	1.63E-02	3.50E-02	1.46E-02
210271_at	NEUROD2	4.07E-01	7.20E-01	2.72E-01	9.85E-01	2.31E-01
225165_at	PPP1R1B**	6.52E-02	4.92E-01	9.18E-01	3.25E-01	3.88E-01
202991_at	STARD3	6.70E-12	4.07E-02	8.87E-04	5.16E-03	1.73E-02
205766_at	TCAP**	1.81E-02	1.29E-01	4.07E-01	3.59E-02	2.53E-01
206793_at	PNMT	4.42E-02	6.06E-01	8.03E-01	5.08E-02	1.16E-01
221811_at	PERLD1	3.66E-11	1.53E-01	1.99E-05	4.81E-02	5.67E-02
216836_s_at	ERBB2	2.42E-02	7.48E-01	1.53E-03	1.54E-01	4.46E-02
224447_s_at	C17orf37	6.84E-02	4.81E-02	1.68E-02	2.20E-01	3.70E-02

210761_s_at	GRB7	3.85E-10	3.33E-01	1.83E-03	8.59E-03	1.94E-02
221092_at	IKZF3	2.11E-02	3.61E-02	5.06E-02	2.29E-01	1.10E-01
231442_at	ZPBP2	3.88E-02	3.96E-01	3.14E-02	2.53E-01	4.13E-03
219233_s_at	GSDML	2.59E-02	1.02E-01	4.92E-01	4.32E-01	7.09E-01
