# Supplementary material for: Complex sense-antisense architecture of TNFAIP1/POLDIP2 on 17q11.2 represents a novel transcriptional structural-functional gene module involved in breast cancer progression
Source: BMC Genomics. 2010 Feb 10;11(Suppl 1):S9. doi: 10.1186/1471-2164-11-S1-S9 (PMC2822537; doi:10.1186/1471-2164-11-S1-S9)
Supplement: Additional file 2 — Survival analysis for the TMEM97/TNFAIP1 (Figure S1) and TMEM199/SARM1 (Figure S2) gene pairs. Description: file contains patients grouping and Kaplan-Meier survival curves for the TMEM97/TNFAIP1 and TMEM199/SARM1 gene pairs in the Uppsala and Stockholm breast cancer cohorts. [file 1471-2164-11-S1-S9-S2.pdf]

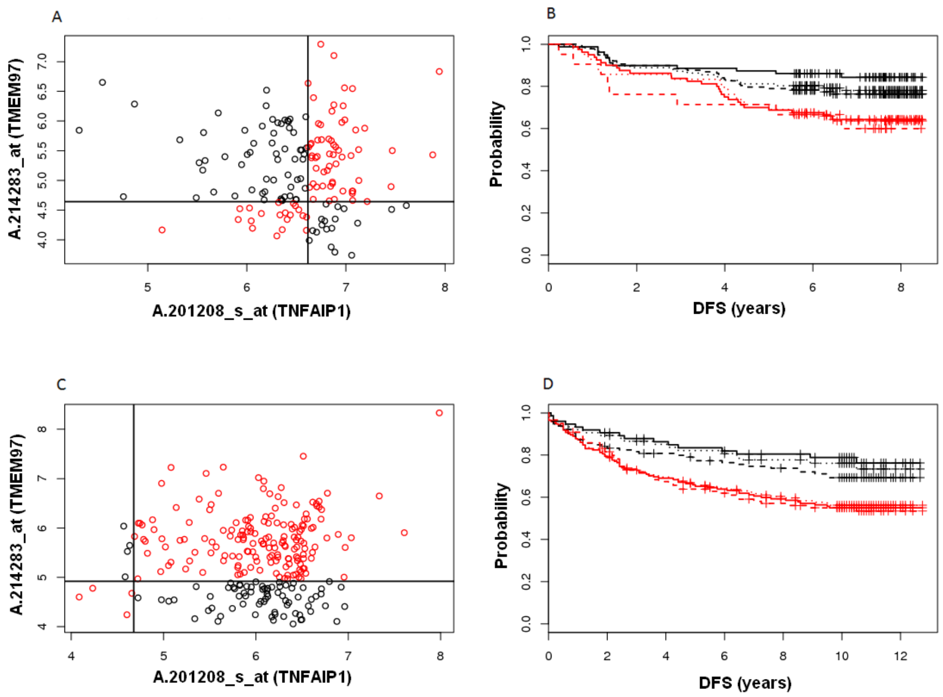

Figure S1. Patients grouping (left) and Kaplan-Meier survival curves (right) in Uppsala (top) and Stockholm (bottom). Patients grouping: Black dots indicate low-risk patients and red dots high risk patients. The horizontal lines are the cut-off of 2D data-driven grouping. Kaplan-Meier curves: The solid lines corresponds to gene synergy, the dashed lines corresponds to TNFAIP1 and the dotted lines toTMEM97.

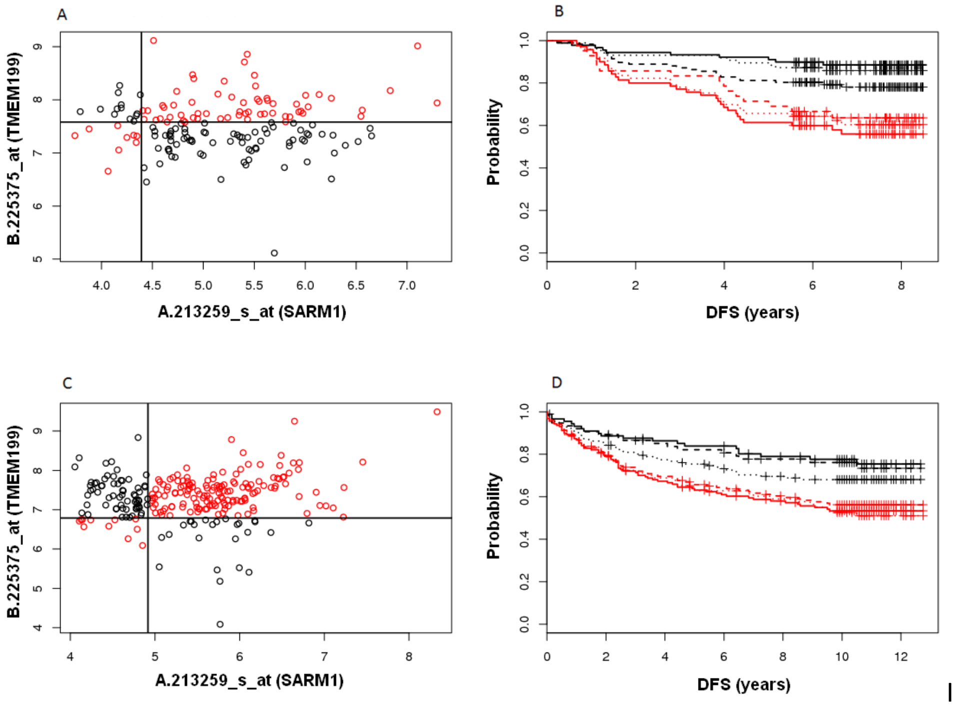

Figure S2. Patients grouping (left) and Kaplan-Meier survival curves (right) in Uppsala (top) and Stockholm (bottom). Patients grouping: Black dots indicate low-risk patients and red dots high risk patients. The horizontal lines are the cut-off of 2D data-driven grouping. Kaplan-Meier curves: The solid lines corresponds to gene synergy, the dashed lines corresponds to SARM1 and the dotted lines to TMEM199.
