# Supplementary material for: Complex sense-antisense architecture of TNFAIP1/POLDIP2 on 17q11.2 represents a novel transcriptional structural-functional gene module involved in breast cancer progression
Source: BMC Genomics. 2010 Feb 10;11(Suppl 1):S9. doi: 10.1186/1471-2164-11-S1-S9 (PMC2822537; doi:10.1186/1471-2164-11-S1-S9)
Supplement: Additional file 4 — Correlation matrix of the genes involved in the ERBB2 CR in breast cancer cell lines. File represents correlation matrix analysis of the genes involved in the ERBB2 CR as well as 11 'neighbouring' genes in a sample of 38 breast cancer cell lines (Kendall-Tau, α = 1%). [file 1471-2164-11-S1-S9-S4.pdf]

AffyProbe	200029_at	228888_at	239224_at	203497_at	213557_at	210271_at	225165_at	202991_at	205766_at	206793_at	221811_at	216836_s_at	224447_s_at	210761_s_at	221092_at	231442_at	219233_s_at	235136_at
Gene Symbol	RPL19	STAC2	FBXL20	PPARBP	CRKRS	NEUROD2	PPP1R1B	STARD3	TCAP	PNMT	PERLD1	ERBB2	C17orf37	GRB7	IKZF3	ZPBP2	GSDML	ORMDL3
RPL19			0.38															
STAC2																		
FBXL20	0.38			0.44	0.33													
PPARBP			0.44		0.66			0.47			0.32		0.31				0.45	0.48
CRKRS			0.33	0.66				0.59	0.45		0.63	0.50	0.48	0.45			0.43	0.60
NEUROD2																		
PPP1R1B								0.33	0.32				0.43	0.42				
STARD3				0.47	0.59		0.33		0.70	0.35	0.69	0.73	0.74	0.69				0.50
TCAP					0.45		0.32	0.70		0.48	0.66	0.54	0.64	0.54				0.51
PNMT								0.35	0.48		0.31		0.35	0.32	0.38			
PERLD1				0.32	0.63			0.69	0.66	0.31		0.79	0.71	0.72				0.50
ERBB2					0.50			0.73	0.54		0.79		0.69	0.72				0.42
C17orf37				0.31	0.48		0.43	0.74	0.64	0.35	0.71	0.69		0.73				0.46
GRB7					0.45		0.42	0.69	0.54	0.32	0.72	0.72	0.73				0.33	0.42
IKZF3										0.38								
ZPBP2																		
GSDML				0.45	0.43									0.33				0.45
ORMDL3				0.48	0.60			0.50	0.51		0.50	0.42	0.46	0.42			0.45	
